# The Effects of Organizational Justice on Positive Organizational Behavior: Evidence from a Large-Sample Survey and a Situational Experiment

**DOI:** 10.3389/fpsyg.2017.02315

**Published:** 2018-01-10

**Authors:** Xiaofu Pan, Mengyan Chen, Zhichao Hao, Wenfen Bi

**Affiliations:** ^1^School of Culture and Social Development Studies, Southwest University, Chongqing, China; ^2^School of Social Work, University of Alabama, Tuscaloosa, AL, United States; ^3^Department of Sociology, Wuhan University, Wuhan, China

**Keywords:** organizational justice, positive organizational behavior, procedural justice, distributive justice, organizational performance

## Abstract

Employees' positive organizational behavior (POB) is not only to promote organizational function but also improve individual and organizational performance. As an important concept in organizational research, organizational justice is thought to be a universal predictor of employee and organizational outcomes. The current set of two studies examined the effects of organizational justice (OJ) on POB of employees with two different studies, a large-sample survey and a situational experiment. In study 1, a total of 2,566 employees from 45 manufacturing enterprises completed paper-and-pencil questionnaires assessing organizational justice (OJ) and positive organizational behavior (POB) of employees. In study 2, 747 employees were randomly sampled to participate in the situational experiment with 2 × 2 between-subjects design. They were asked to read one of the four situational stories and to image that this situation happen to the person in the story or them, and then they were asked to imagine how the person in the story or they would have felt and what the person or they subsequently would have done. The results of study 1 suggested that OJ was correlated with POB of employees and OJ is a positive predictor of POB. The results of study 2 suggested that OJ had significant effects on POB and negative organizational behavior (NOB). Procedural justice accounted for significantly more variance than distributive justice in POB of employees. Distributive justice and procedural justice have different influences on POB and NOB in terms of effectiveness and direction. The effect of OJ on POB was greater than that of NOB. In addition, path analysis indicated that the direct effect of OJ on POB was smaller than its indirect effect. Thus, many intermediary effects could possibly be between them.

## Introduction

Employee motivation and organizational effectiveness are the eternal topics of enterprise human resource management. The human resource management in an enterprise is finding ways to achieve the ultimate goal of inspiring employee motivation. Therefore, the study of employees' positive organizational behavior (POB) is attracting more and more attention. Luthans (2002a,b) introduced the theory of positive psychology to the field of organizational behavior, and defined POB as “the study and application of positively oriented human resource strengths and psychological capacities that can be measured, developed, and effectively managed for performance improvement in today's workplace” (2003, p. 179). Based on the Chinese cultural background, (Pan, 2008), and Pan and Qin (2009) defined POB as organizational behavior of employees which are beneficial to organizations. It can promote organizational function as well as improve individual and organizational performance. They also identified six dimensions of POB: devoted, responsible, active, innovative, helping, and harmonious behaviors.

Several studies have been conducted in order to find the reasons behind the employees' willingness to show their POB. There are several factors including personal traits (Youssef and Luthans, 2007; Uymaz, 2014; Leephaijaroen, 2016), job characteristic (Bakker and Schaufeli, 2008), work stressors (Munir, 2013), and economic situation (Giorgi et al., 2015; Mucci et al., 2016; Lopez-Valcarcel and Barber, 2017). Giorgi et al. (2015) suggested that during the economic crisis, employees are more likely to have an ambiguous view of their role in organizations or a perception that hard work is more stressful as they would not get fruitful benefits. These thoughts may negatively affect their emotional and behavioral outcomes for organizations, such as less cooperative. However, what worth mentioning are these aspects described above should not be viewed as a comprehensive, exhaustive explanation of what influences POB of employees. The study of POB needs further discussion as there may have better explanatory factors affecting POB of employees.

According to Organs view (Organ, 1990), an organizational member's decision to behave may be a function of the degree to which an employee believes that he or she has been treated fairly by the organization. Previous researches showed that organizational justice is associated with different positive organizational outcomes. For example, Wang et al. (2010) suggested that organizational justice can help improve the employees' work performance. Demirkiran et al. (2016) showed that if employees perceive that actions and practices in the organization are fair and honest, they will show more extra-role behavior, which is beneficial to the development of organizations. Saifi and Shahzad (2017) found that positive perception of employees in relation to organizational justice is an important antecedent to employees' job satisfaction, which in turn promote positive behavior of employees. On the other hand, researches suggested that employees may respond to perception of unfair treatment with a range of negative behavioral responses (e.g., theft, withdrawal, resistance, vandalism, sabotage, and reduction of positive behavior; Fox et al., 2001; Lilly, 2017).

Therefore, the purpose of this study was to examine the relationship between organizational justice and POB of employees, and whether different dimensions of organizational justice can have different impacts on POB and NOB of employees.

## The theoretical background and hypothesis

### Organizational justice

Justice is recognized as an action or decision that is understood to be morally right on the basis of ethics, religious, fairness, equity, or law (Pekurinen et al., 2017). It is a major area of concern for both organizations and employees (Swalhi et al., 2017). Organizational justice refers to employee's perception of fairness within an organization (Greenberg, 1990; Asadullah et al., 2017).

The earliest idea of organizational justice was derived from equity theory (Adams, 1963, 1965). It suggested that people compare the ratios of their own perceived work outcomes to their own perceived work inputs with the corresponding ratios of their counterparts. So, their organizational participation can be changed (Colquitt et al., 2001). Input here refers to time and effort and output refers to rewards, such as promotion, pay, recognition, equipment, or any other job-related resources that assist employees in job tasks or maintain overall well-being (Ghosh et al., 2017). If the ratios are equal, people in the organizational contexts are expected to have equitable and satisfied feelings. However, if the ratios are unequal, employees may have the feeling of injustice, they would try to change the situation to create new balance. For example, they may choose to reduce their input-output comparison (Shkoler and Tziner, 2017). Furthermore, organizational justice is also rooted in social exchange theory, which treats social life as a series of sequential transactions between two or more parties (Blau, 1964). In these transactions, resources are exchanged through a process of reciprocity. Therefore, one party tends to repay the good (or sometimes bad) deeds of another party (Cropanzano et al., 2017). Work relationship can be seen as a form of transaction. For example, someone exchanges work for income (Cropanzano et al., 2002). Employee's perception of justice determines the quality of exchanging relationship with organization (Swalhi et al., 2017). When employees perceive fair treatment from the organization and its authorities, they may feel a sense of obligation to create a good act in return (Ghosh et al., 2017).

A number of studies suggested that organizational justice is a key cause of many factors which affect employees' attitudes (e.g., job satisfaction, turnover intentions, and organizational commitment) and behaviors, such as innovative work behavior, organizational citizenship behavior as well as work performance. For example, Usmani and Jamal (2013) examined the relationship between organizational justice and job satisfaction and found that distributive justice, interactional justice and personal time are positively related to job satisfaction. Employees are willing to do more work and exhibit higher levels of performance when they believe they are treated fairly (Köse, 2014). Akram et al. (2016b) suggested that organizational justice has a strong and positive impact on the innovative work behavior of the Chinese employees. Swalhi et al. (2017) demonstrate that organizational justice affects the behavior and performance of employees in the some small-and medium-sized enterprises (SMEs). Studies also showed that justice perceptions have a robust link with organizational citizenship behavior (Karriker and Williams, 2009; Tziner and Sharoni, 2014; Gurbuz et al., 2016). When perception of organizational justice is high, it can enhance employees' positive attitudes toward their organizations and OCB (Özbek et al., 2016). Nevertheless, low level of organizational justice would lead to dissatisfaction and negative feelings of employees, which, in turns, lead to some negative consequences. For example, Pekurinen et al. (2017) stated that low organizational justice may has an adverse effect on nurses' behavior toward colleagues (e.g., collaboration) and may lead to poor employee-patient interactions and change nurses' behavior toward patients. Shkoler and Tziner (2017) shown that the perception of injustice can pose a threat to employees' resources and give them a feeling of inappropriate resources. It makes them feel frustrated and even wear them out, which, in turn, evolve into burnout and destructive organizational behaviors, such as theft, sabotage, withdrawal, harassment.

In developing the theory of organizational justice, researchers have identified three main models including (a) two-factor model, namely distributive and procedural justice; (b) three-factor model, namely distributive, procedural and interaction justice; (c) four-factor model, namely distributive, procedural, interpersonal, and informational justice. Although many existing researches studied organizational justice by using the three-factor or four-factor model (Cohen-Charash and Spector, 2001; Colquitt et al., 2001; Tessema et al., 2014; Akram et al., 2016a,b), there is less agreement about the distinction between the interactional justice and procedural justice, informational justice and interactional justice due to the high inter-correlation (Colquitt et al., 2001). Hence, it is currently unclear that whether organizational justice should be divided by the three or four factors. Nevertheless, it must be noted that researchers have reached an agreement regarding the distinction between the procedural and distributive justice (Tessema et al., 2014). The two-factor model is the most common model used to analyze organizational justice (Alexander and Ruderman, 1987; Moorman, 1991; McFarlin and Sweeney, 1992; Karriker and Williams, 2009; Strom et al., 2014; Ghosh et al., 2017) and also serves as a baseline for the following three-, four-factor models. Each of the justice factors is briefly discussed as below.

### Distributive justice

Distributive justice denotes the perceived fairness of the outcomes received by an employee (Moorman, 1991). Lawler suggested that these outcomes, such as pay, promotion, status, performance evaluations, and job tenure would have great influences on job satisfaction, quality of work life, and organizational effectiveness (Alexander and Ruderman, 1987). It is the equity theory that guides the outcome-oriented viewpoint. Adams conceptualized distributive justice (Tessema et al., 2014) and claimed that people are concerned about whether the outcomes are fair instead of the absolute level of the outcomes (Colquitt et al., 2001). When an outcome is perceived to be unfair, it can affect individual's emotion (e.g., anger, happiness, pride, or guilt) and cognitions (e.g., cognitively distort inputs and outcomes of himself/herself or of the other) as well as their behavior (e.g., performance and withdrawal; Cohen-Charash and Spector, 2001). Campbell et al. (2013) suggest that the perception of distributive justice is associated with the allocating resources. In other words, the feeling of fairness depends on such a way that employees perceived that resources have been shared equitably and replenished adequately. A number of studies suggested that distributive justice and procedural justice have different impacts on organizational outcomes. For example, McFarlin and Sweeney (1992) utilized a main effect approach to examine the predictive roles of distributive and procedural justice and found that distributive justice tends to be a stronger predictor of personal outcomes (e.g., pay level satisfaction, and job satisfaction). Fields et al. (2000) found that distributive justice has larger effects on Hong Kong employees' intent to stay and job satisfaction, but procedural justice plays a more important role in determining Hong Kong employees' evaluation of supervision. Cropanzano et al. (2002) suggested that distributive justice tends to strongly correlate with reactions to specific outcomes and less strongly correlate with reactions to the organization or to one's supervisor. Ghosh et al. (2017) found that distributive justice is a stronger predictor of the sacrifice dimension of organizational embeddedness than procedural justice.

### Procedural justice

Procedural justice refers to “the individual's perception of fairness of procedural elements within a social system regulates allocation of resources” (Leventhal, 1980). It fits with the final outcomes that are equitably deal with methods, mechanisms, and processes (Swalhi et al., 2017). It is considered to exist when procedures embody certain types of normatively accepted principles. Specifically, the fairness of the procedures shall meet the following criteria: the extent to which they suppress bias, create consistent allocations, rely on accurate information, are correctable, represent the concerns of all the recipients, and are based on the prevailing moral and ethical standards (Leventhal, 1980).

In the setting of organizations, procedural justice is considered as the root of social exchange (Swalhi et al., 2017). It has a significant impact on employees' cognitive, affective, and behavioral reactions toward the organization (Cohen-Charash and Spector, 2001). For example, Cropanzano et al. (2002) suggested that procedural justice is more likely associated with trust in upper management and organizational commitment. Kim and Park (2017) stated that procedural justice positively influences employee's work engagement, knowledge sharing and innovative work behavior. Lee et al. (2017) showed that procedural justice can facilitates employees to accept the change of values and objectives of organization and also adapt themselves to pressures of external change. Furthermore, certain findings suggested that the process of allocating rewards is more important than the result (Lind and Tyler, 1988; Cohen-Charash and Spector, 2001).

### Positive organizational behavior

POB stems from positive psychology which was led primarily by Seligman and other well-known positive psychologists (Wright, 2003). Seligman and Csikszentmihalyi (2014) suggested that the purpose of positive psychology is “to begin to catalyze a change in the focus of psychology from repairing the worst things in life to building positive qualities.” Therefore, positive psychology primarily studies individuals' strengths and virtues that are beneficial to the development of individuals and communities (Bakker and Schaufeli, 2008). Following the lead of positive psychology, Luthans (2002a) perceived the need for a new theoretical and research-driven perspective and approach to the organizational research, which he termed POB, that is “the study and application of positively oriented human resource strengths and psychological capacities that can be measured, developed, and effectively managed for performance improvement in today's workplace” (Youssef and Luthans, 2007). Specifically, a positive psychological capacity which can be included into the POB framework must be positive and must have theory and research back-up as well as valid measures. Furthermore, this capacity should make it open to any change and development (i.e., state-like) and have relation to performance improvement in the workplace (Luthans, 2002b). The six positive psychological capacities, namely confidence (or self-efficacy), hope, optimism, resilience, subjective well-being (or happiness), and emotional intelligence specifically meet the definition of POB and inclusion criteria, and are viewed as a contribution to understand POB and have considerable impacts on organization performance (Luthans, 2002b; Youssef and Luthans, 2007). However, Wright (2003) counterbalanced this utilitarian and management-driven view as well as the focus on organization instead of individuals, and argued that the objective of POB should also include the pursuit of employee happiness and health as viable goals in themselves. He introduced Fredrickson's broaden-and-build model which suggests the potentially adaptive and interactive nature of positive emotions (Wright, 2003). According to Fredrickson (2002), the adaptive or moderating nature of such positive emotions as happiness and joy is potentially more robust for those who are more joyous than for those who are less joyous. He suggested that such positive impetus can enable people to be more creative, resilient, socially connected, and physically and mentally healthy (Wright, 2003). Bakker and Schaufeli (2008) proposed that the organization-based perspective of Luthans and the employee-based perspective should be integrated; POB should emphasize on individual positive psychological conditions and human resource strengths that are relevant to both performance improvement and employees' well-being.

But it should be noted that these researches related to POB are normally concentrated on the implicit and psychological constructs of POB, has not yet attached with importance to the explicit form of positively oriented human resource strengths and psychological capacities. Accordingly, Pan (2008), based on the Chinese cultural background, proposed a new perspective of employees' POB, which could be defined as employees' positive behavior in organization. They proposed that employees' POB is mainly composed of devoted, responsible, active, innovative, helping, and harmonious behavior (Pan and Qin, 2009). Employees' POB can not only to promote organizational function but also improve individual and organizational performance. In this study, employees' POB consisting of devoted, responsible, active, innovative, helping, and harmonious behavior was considered as the dependent variable, while organizational justice was regarded as the independent variable.

### Relationship between OJ and POB

Organizational justice is found to be a key factor of many organizational outcome variables, such as trust, commitment, job satisfaction, organizational citizen behavior, job performance, and POB (Alexander and Ruderman, 1987; Moorman, 1991; McFarlin and Sweeney, 1992; Cohen-Charash and Spector, 2001; Colquitt et al., 2001; Wong et al., 2006; Karriker and Williams, 2009; Zainalipour et al., 2010; Keyvanar et al., 2014; Khan et al., 2016; Nastiezaie and Jenaabadi, 2016). For instance, Alexander and Ruderman (1987) suggested that all fairness variables, as a group, are significantly associated with employees' work-related attitudes and behaviors and procedural fairness and distributive fairness have distinct effects on the organizational outcomes. Keyvanar et al. (2014) studied organizational justice and POB in the context of hospital and found that organizational justice is related to POB (hope, optimism, self-efficacy, and resiliency) and work engagement through the attainment of personal career goals. Nastiezaie and Jenaabadi (2016) showed that organizational justice has a significant and positive correlation with POB A small number of researches explored how perceptions of fair treatment influence the employee's beneficial behavior. For example, Joseph et al. (2015) found that organizational justice had a significant effect on interpersonal helping behavior. Walumbwa et al. (2009) examined the relationship between organizational justice and voluntary learning behavior, and found that perceptions of employee distributive and procedural justice had an indirect impact on learning behavior. These studies all suggested that organizational justice and positive behavior in organization have certain correlation, and organizational justice would have a significant impact on employees' positive behavior. By contrast, employees treated with organizational injustice might perform negative behavior. For example, DeMore et al. (1988) found that low perceived equity (lack of fairness in one's social or environmental arrangements) can predict vandalism. Ambrose et al. (2002) examined the relationship between injustice and workplace sabotage, and found that injustice is the most common cause of sabotage. Min et al. (2014) suggested that perceived injustice during work is significantly associated with an increased risk of occupational disease and absenteeism for Korean employees. Mingzheng et al. (2014) suggested that organizational justice is negatively correlated with counterproductive work behavior among Chinese public servants. Finding from Michel and Hargis (2017) showed that procedural injustice motivates deviant behavior in the workplace.

Based on these considerations, we expect that organizational justice and POB of employees will have a significant relationship, and different dimensions of organizational justice will lead to different behavioral outcomes. Thus, we want to investigate the relationship between POB and OJ and how distributive justice and procedural justice will affect the POB of employee and negative organizational behavior (NOB).

### Hypotheses

In view of the above, four hypotheses are proposed as the following:

H1: If OJ is positively related to POB, then employees with a high level of OJ will perform more POB.
H2: If OJ is a positive predictor of POB, then higher level of OJ will predict higher level of POB.
H3: OJ was expected to have a significant main effect on employees' POB.
H4: If procedural justice differs from distributive justice in terms of influence effectiveness and direction, then procedural justice and distributive justice will have different influences on employees' POB and NOB.

## Overview of studies

Justice theory states that the perception of the employees about fairness leads to certain reactions (positive or negative), and in turns leads to certain behavior (positive or negative; Akram et al., 2016b). Specifically, the perceived justice can motivate employees to perform more beneficial and positive behavior for organizations, while, when experiencing injustice they might react negatively (Graso and Grover, 2017). A substantial body of empirical work demonstrates that organizational justice have significant impact on employees' behavior, and distributive justice and procedural justice can distinctly influence employees' work-related attitudes and behavioral outcomes (Cohen-Charash and Spector, 2001). Therefore, we employed a large-sample survey and a situational experiment to examine the effect of OJ in the form of distributive justice and procedural justice on POB and NOB among enterprise employees. In study 1, we attempted to analyze the relationship between OJ and POB among enterprise employees through a survey study in which participants were then asked to report their level of organizational justice (OJ) and positive organizational behavior (POB) with self-made valid scales. In study 2, we attempted to further findings from Study 1 through a situational experiment with 2 × 2 between-subjects design in which participants were asked to read one of the four situations stories and to imagine that this situation happen to either the person in the story (Evaluate by the situation) or them (Evaluated by self-experience), and then they were asked to imagine how the person in the story or they would have felt and what the person or they subsequently would have done. Specifically, in situational experiment organizational justice including distributive justice and procedural justice would be reflected in two aspects (justice and injustice), and the outcome variables include POB and NOB of employees. We examined whether procedural justice differs from distributive justice in terms of effectiveness and direction of effect on POB and NOB of employees.

## Study 1

### Methods

#### Participants and procedure

From 13 cities in China, a total of 2,566 employees were randomly selected from 45 manufacturing-type enterprises. Male employees accounted for 44.7% and females accounted for 55.3%. Respondents aged under 25 accounted for 30.7%, 25–34 years old accounted for 35.9%, 35–44 years old accounted for 22.8%, 45–54 years old occupied 8.7%, and 55 years old and above took up for 1.9%. Respondents graduating from high school and below accounted for 62.4%, with junior college degree accounted for 24.9%, with bachelor degree accounted for 11.3%, and with master's and Ph.D. degree occupied 1.4%. In addition, ordinary employees accounted for 60.5%, first-line managers accounted for 24.2%, middle managers accounted for 10.4%, and senior managers occupied 4.8%. Respondents with < 1 year work experience accounted for 14.2%, with 1–2 years work experience took up for 32.8%, with 3–5 years work experience accounted for 22.6%, with 6–10 years work experience occupied 11.7%, and with over 10 years work experience accounted for 14.6%. Respondents who received a monthly salary of ¥2,000 accounted for 25.4%, received a monthly salary of ¥2,001 to ¥3,500 accounted for 51.1%, received a monthly salary of ¥3,501 to ¥5,000 accounted for 13.7%, and those who received ¥5,000and above accounted for 9.8%. This study received ethics approval from the University of Southwest's Human Research Ethics Committee'. All participants were informed that participation was purely voluntary. No payments were offered in exchange for participation. After providing the written informed consent, participants completed two self-made questionnaires. In order to minimize common method bias, we firstly assured the anonymity and confidentiality of all survey responses by tracking data with site coding rather than respondents names and having surveys returned directly to the researchers. Secondly, we designed the response questionnaire with A and B columns (column A—for any one company; column B—for your company), reflecting the combination of self-evaluation and other-rated method, to reduce potential social desirability. Subsequent analyses suggested that the difference between A and B was not significant (*t* = 1.826, *P* > 0.05), the social desirability effects were deemed small. Thirdly, we utilized the pre-survey with a small sample of 368 employees from 12 companies, and 3 months later in the formal investigation these participants were again asked to complete the same questionnaire. Subsequent analysis suggested that there is no significant difference between these two survey outcomes (*t* = 1.912, *P* > 0.05). Additionally, we adopted other ways to minimize the effect of non-related variables on the survey outcomes such as training investigators, using the unified instruction and trying to control the effect of the situational factors.

#### Measures

##### Organizational justice

According to Joy and Witt's (1992) theory that organizational justice can be divided into distributive and procedural justice, we developed a 12-item scale as an instrument for measuring organizational justice. Because the set of 12 items tapped different aspects of organizational justice, we carried out EFA to identify any underlying dimensions. The exploratory factor analysis (EFA) yielded two factors that explained 72.11% of the common variance. For the distributive justice factor, a measure consisting of 5 items (factor loading range from 0.672 to 0.836) was constructed. For the procedural justice factor, a measure consisting of three items (factor loading range from 0.818 to 0.843) was constructed.

Further, to take into consideration organizational justice in its entirety, we conducted confirmatory factor analysis (CFA) in which all the organizational justice items were loaded onto their respective factors. The results showed a good fit (χ^2^/df = 7.68, GFI = 0.97, NFI = 0.99, RFI = 0.98, IFI = 0.99, CFI = 0.99, RMSEA = 0.075 and SRMR = 0.024) and the coefficient alpha was 0.913.

##### Positive organizational behavior (POB)

Employees' POB was measured by using the scale developed and validated by Pan (2008), Pan and Qin (2009). This scale consists of 33 items loading on six distinct factors, which include devoted behavior (employees devote their time and energy to their work), responsible behavior (employees complete their work voluntarily), active behavior (employees can adapt to the external environment willingly), innovative behavior (employees are willing to embrace new technologies and apply or create new technology at work), helping behavior (employees help colleagues complete work willingly), and harmonious behavior (employees cooperate with others in a friend way). The EFA yielded six factors that explained ~70% of the common variance. The results of CFA showed a good fit (χ^2^/df = 3.96, RMSEA = 0.065, GFI = 0.86; NFI, NNFI, CFI, IFI, RFI, and TLI were above 0.95). The coefficient alpha was 0.97 and the retest reliability was 0.88 (*r* = 0.88).

### Result

To verify the validity of the hypotheses proposed in the current study, we used SPSS 20.0 and LISREL8.7 to analyze the obtained data.

#### Correlation analysis

Table 1 reports the means, standard deviations and correlation coefficients between all the variables in the current study.

**Table 1:** Correlation analysis (*n* = 2566).

	**M ± SD**	**1**	**2**	**3**	**4**	**5**	**6**	**7**	**8**	**9**	**10**
1. DB	3.25 ± 0.74	1									
2. RB	5.07 ± 0.59	0.36^**^	1								
3. AB	3.79 ± 0.63	0.51^**^	0.66^**^	1							
4. IB	3.74 ± 0.66	0.48^**^	0.61^**^	0.79^**^	1						
5. Help-B	3.63 ± 0.70	0.50^**^	0.51^**^	0.69^**^	0.77^**^	1					
6. Harm-B	3.81 ± 0.67	0.42^**^	0.55^**^	0.67^**^	0.74^**^	0.78^**^	1				
7. PJ	3.18 ± 1.15	0.31^**^	0.26^**^	0.34^**^	0.36^**^	0.39^**^	0.36^**^	1			
8. DJ	3.40 ± 0.95	0.35^**^	0.32^**^	0.38^**^	0.41^**^	0.43^**^	0.39^**^	0.75^**^	1		
9. OJ	5.58 ± 1.97	0.35^**^	0.31^**^	0.38^**^	0.41^**^	0.45^**^	0.40^**^	0.95^**^	0.92^**^	1	
10. POB	3.71 ± 0.54	0.68^**^	0.74^**^	0.87^**^	0.89^**^	0.87^**^	0.85^**^	0.41^**^	0.47^**^	0.47^**^	1

*n = 2566*.

** *p < 0.01, Two tailed test; DB, Devoted behavior; RB, Responsible behavior; AB, Active behavior; IB, Innovative behavior; Help-B, Helping behavior; Harm-B, Harmonious behavior; PJ, Procedural justice; DJ, Distributive justice*.

These tests are based on the scores from the scales previously mentioned. Overall POB and OJ were calculated according to the scores of its own dimensions. The results show that there are numerous significant positive correlations between all the variables. H1 was therefore accepted.

#### Multiple regression analysis

For testing the casual effect of distributive justice, procedural justice and overall OJ on employee devoted, responsible, active, innovative, helping, harmonious behavior, and overall POB, a number of models were developed by multiple linear regression analysis.

As shown in the Table 2, the results revealed that the regression equation established by the two factors of OJ and all factors of staff POB had significant statistical significance (each *F-*value's *p* < 0.001). Moreover, the procedural justice and distributive fairness had very significant positive effect on various factors of employees' POB. Procedural justice and distributive justice commonly explained investment, responsible, initiative, innovation behavior, helping, and harmonious behaviors by 14, 11, 17, and 18, 22, and 18% of variation, respectively. Moreover, the results show that overall OJ is a positive predictor (β = 0.51, *p* < 0.001, *R*^2^ = 0.26) of overall POB. H2 could be proved.

**Table 2:** Regression analysis of OJ on POB (*n* = 2566).

**Dependent variable**	**Argument**	***Beta***	***T***	***R***	***R^2^***	***$R_{adj}^{2}$***	***F***
Devoted behavior	Procedural justice	0.11	3.74^**^	0.37	0.14	0.13	187.31^**^
	Distributive justice	0.28	9.67^**^				
Responsible behavior	Procedural justice	0.06	2.799^*^	0.33	0.11	0.10	149.57^**^
	Distributive justice	0.29	9.90^**^				
Active behavior	Procedural justice	0.15	5.97^**^	0.41	0.17	0.14	228.52^**^
	Distributive justice	0.28	9.92^**^				
Innovative behavior	Procedural justice	0.13	5.539^**^	0.43	0.18	0.17	281.79^**^
	Distributive justice	0.33	11.96^**^				
Helping behavior	Procedural justice	0.17	5.82^**^	0.47	0.22	0.19	321.55^**^
	Distributive justice	0.34	11.87^**^				
Harmonious behavior	Procedural justice	0.18	5.77^**^	0.42	0.18	0.15	249.81^**^
	Distributive justice	0.29	9.81^**^				
POB	Procedural justice	0.16	5.72^**^	0.49	0.24	0.22	381.93^**^
	Distributive justice	0.37	13.47^**^				
	OJ	0.51	25.74^**^	0.51	0.26	0.21	719.85^**^

* p < 0.05;

** *p < 0.01, two tailed test*.

#### Path analysis

**Figure 1:**
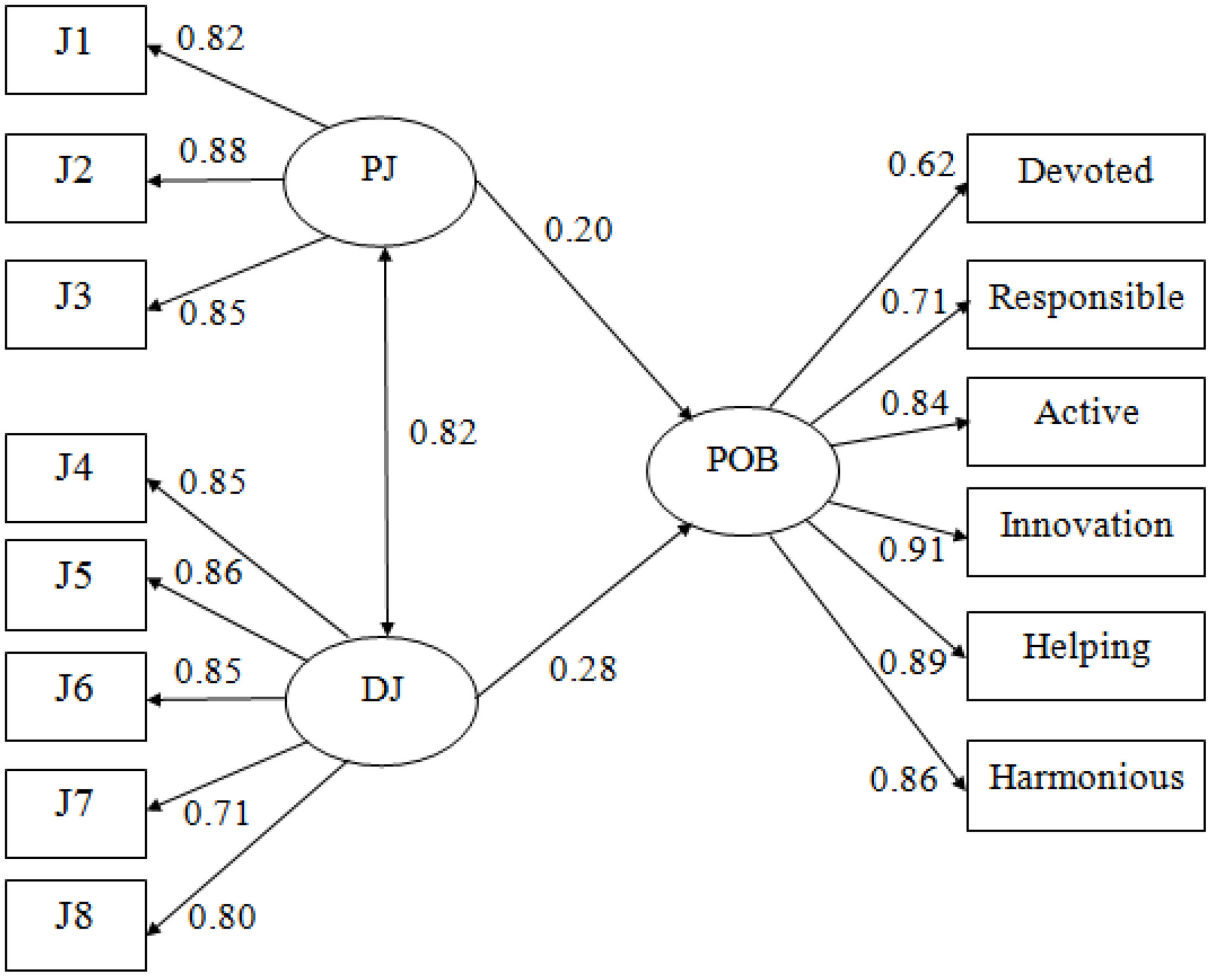
Standardized path coefficients of the effect of OJ on POB. PJ, Procedural justice; DJ, Distributive justice; J1~J8, items of the scales of Organizational Justice.

In Table 3 and Figure 1, path analysis can allow us to examine the direct, indirect, and total effect between the analysis variables. The results show that the total effects of distributive justice and procedural justice on all dimensions of POB of employee were significant. Specifically, the total effects of distributive justice on devoted behavior and responsible behavior were strongest (β = 0.55, *t* = 37.35, *p* < 0.001 and β = 0.50, *t* = 31.99, *p* < 0.001, respectively), and procedural justice was the strongest predictor of active behavior of employee (β = 0.36, *t* = 21.72, *p* < 0.001). By contrast, the overall effects of distributive justice and procedural justice on helping behavior of employee were relatively low (β = 0.17, *t* = 8.59, *p* < 0.001 and β = 0.25, *t* = 12.53, *p* < 0.001, respectively).

**Table 3:** Path analysis.

**Variable relations**	**Direct effect beta (t)**	**Indirect effect beta (t)**	**Total effect beta (t)**
ξ1 → η1	0.31 (21.35)^**^	–	0.31 (21.35)^**^
ξ1 → η2	0.10 (7.26)^**^	0.20 (18.56)^**^	0.30 (19.05)^**^
ξ1 → η3	0.14 (9.91)^**^	0.22 (18.43)^**^	0.36 (21.72)^**^
ξ1 → η4	−0.02 (-1.64)	0.28 (20.96)^**^	0.26 (15.05)^**^
ξ1 → η5	0.13 (5.21)^**^	0.12 (10.64)^**^	0.25 (12.53)^**^
ξ1 → η6	0.07 (5.27)^*^	0.20 (13.20)^**^	0.27 (13.95)^**^
ξ2 → η1	0.55 (37.35)^**^	–	0.55 (37.35)^**^
ξ2 → η2	0.15 (9.76)^**^	0.35 (25.49)^**^	0.50 (31.99)^**^
ξ2 → η3	0 (0.10)	0.38 (25.86)^**^	0.38 (22.65)^**^
ξ2 → η4	0.11 (5.85)^**^	0.35 (23.35)^**^	0.46 (27.03)^**^
ξ2 → η5	0.01 (0.56)	0.16 (9.76)^**^	0.17 (8.59)^**^
ξ2 → η6	0.06 (3.57)^*^	0.16 (9.21)^**^	0.22 (11.53)^**^

ξ1, Procedural justice; ξ2, Distributive justice; η1, Devoted behavior; η2, Responsible behavior; η3, Active behavior; η4, Innovative behavior; η5, Helping behavior; η6, Harmonious behavior;

* *P < 0.05*,

** *P < 0.001, beta: Standardized regression coefficient t: t-test value*.

Furthermore, the direct effects of distributive justice and procedural justice on all dimensions of POB of employee were quite low. Particularly, distributive justice failed to directly affect innovative behavior, and procedural justice failed to directly affect active, helping behavior. However, it should be noted that distributive justice and procedural justice have significant and positive indirect effects on all dimensions of POB of employee. Hence, we can conclude that the relationships among distributive justice and innovative, procedural justice and active behavior, and procedural justice and helping behavior were mediated by other variables. Thus, these results provide support for H3.

## Study 2

### Materials and methods

#### Participants

In the pre-test, we randomly selected 96 employees from three manufacturing-type enterprises located in Chenzhou, China. These subjects were equally divided into four groups with 24 subjects in each group. Each group was randomly assigned to a situation. The pool of subjects included 51 male and 45 female employees, and their average age was 36.31 years old. Thirty one were managers and 65 were ordinary employees. The number of people with the degree above junior college was 63.

In the formal experiment, a total of 800 employees were randomly selected from 16 manufacturing-type enterprises located in six cities of Hunan, Guangdong, and Zhejiang province, China. Similarly, these were equally divided into four groups with 200 subjects in each group. Each group was randomly assigned to a situation. Finally, a total of 747 effective samples were obtained. Among them, 191 were effective samples for situation 1 (A1B1), 177 for situation 2 (A1B2), 189 for situation 3 (A2B1), and 190 for situation 4 (A2B2). The pool of subjects included 418 men (56%) and 329 women (44%). In this sample, 25 years old and below accounted for 30.1%, 25–34 years old 37.7%, 35–44 years old 22.9%, 45–54 years old 6.9% and 55 years old and above 2.3%. In terms of level of education, 52.7% of respondents graduated from high schools and below, 26.8% of respondents held a junior college degree, 17.7% of respondents held an bachelor degree and 2.7% of respondents held a master's and Ph.D. degree. In addition, ordinary employees accounted for 64.4%, first-line managers 22.3%, middle managers 10.8% and senior managers 2.5%.

This study was carried out in accordance with the recommendations of “the University of Southwest's Human Research Ethics Committee” with written informed consent from all subjects. All subjects were purely voluntary, and gave the written informed consent. No payments were offered in exchange for participation.

#### Experimental materials

Before the experiment, the research interviewed the participants about the bonus issues, and gathered the typical cases of distributive justice and injustice and procedural justice and injustice in the process of bonus distribution. After refining these typical cases, four situational stories on bonus distribution were designed as experimental materials. These four situational stories respectively represented four type of experimental treatments, which included A1B1 (distributive justice × procedural justice); A1B2 (distributive justice × procedural injustice); A2B1 (distributive injustice × procedural justice); and A2B2 (distributive injustice × procedural injustice). Each story was in accordance with the logic of the event development, which means that the bonus distribution was conformed to the order from the process to the outcomes.

This is an example of the situational story one. (A1B1: distributive justice × procedural justice). The situational stories of A1B2, A2B1, and A2B2 are shown in the Appendix.

(Story) *Senior managers of a company intend to give a large amount of bonus for employees. They formulate the standards and organize managers of each layer and representative of the employees to have a discussion. After discussions, the distributive standard is determined preliminarily. Then, the document of the standard is shown publicly to collect opinions of ordinary employees until the document is approved by all staff. Based on the arrangement without objection, the Personnel Department evaluates every employee according to the distributive document and personal job performance. The result is shown publicly for correction of mistakes. According to the distributive arrangement and personal job performance, Zhangsan obtained the lowest score and the minimum bonus*.

(Instructions)Please answer the following questions based on your real thoughts and physical truth according to the situational story. When answering the questions, mark directly on the selected option (5 = Absolutely agree, 4 = Partly agree, 3 = A little bit agree, 2 = Partly disagree, and 1 = Absolutely disagree).

Absolutely disagree—Absolutely agree
*(1) Based on the bonus distributive procedure mentioned above, Zhangsan will think it is fair and he will do his work actively*.
1 2 3 4 5
*(2) Based on the bonus distributive procedure mentioned above, if we were employees in the company, we will also feel it is fair and do our work actively*.
1 2 3 4 5
*(3) Based on the bonus distributive procedure mentioned above, Zhangsan will feel it is unfair. He will slow down, be absent, not obey the arrangement in his work, or even resign*.
1 2 3 4 5
*(4) Based on the bonus distributive procedure mentioned above, if we were employees in the company, we will also feel it is unfair. We will slow down, be absent, not obey the arrangement in our work, or even resign*.
1 2 3 4 5

#### Design

The situational experiment used a 2 × 2 between-subjects design. The independent variables were organizational justice in form of distributive justice and injustice and procedural justice and injustice. The specific operational definition of these independent variables as follows: (1) Distributive justice: More labor efforts, higher production rate, and more contributions result in higher bonus. By contrast, less labor effort, lower production rate, and less contribution resulted in lower bonus; (2) Distributive injustice: More labor effort, higher production rate, and more contributions result in lower bonus. By contrast, less labor effort, lower production rate, and less contribution led to higher bonus. (3) Procedural justice: Bonus distribution standard justice, process-transparent, accurate information, publicly showed result, and correctable mistakes; (4) Procedural injustice: Injustice bonus distribution standard, closed procedure, inaccurate information, and closed results. The response variables are POB and NOB of employees. POB here refers to the devoted, active, helping, responsible, innovative and harmonious behavior. NOB refers to inimical, aggressive, and backward-looking behavior, and mainly performs as discontentment, hostility, sabotage, absence, and retirement. The response variables were measured by two types of indexes: (1) Evaluated by the situation (The subject was asked to give a response to the experience of hero in the story) and (2) Evaluated by self-experience (the subject was asked to read the situational story carefully and then answer the following questions according to his real thoughts assuming that he is the hero in the story). The scores of these two types of indexes both adopted a five-point Likert scale, where 1 indicates disagree absolutely and 5 indicates agree absolutely. Additionally, we controlled for age, gender, level of education, and organizational position to rule out possible alternative explanations for our findings.

#### Procedure

At the beginning of this study, the examiners explained to each subject about the nature and the aim of the manipulation and ensured all responses would be kept confidential and anonymous with the same instruction and same situational condition. Subsequently, examiners randomly assigned one certain situational story to each group and asked the subjects to read the story carefully and then make judgment: (a) choosing the best answers to the following questions according to the feelings of the person in the story—Evaluated by the situation; (b) choosing the best answers to the following questions according to their own feeling supposing themselves as the person in the story—Evaluated by self-experience.

### Results

#### Correlation analyses

The means, standard deviations, and correlations among the study variables are displayed in Table 4. The results show that distributive justice and procedural justice were correlated with POB, and further the correlation between procedural justice and POB (*r* = 0.319, *p* < 0.01) was greater than between distributive justice and POB (*r* = 0.079, *p* < 0.05). Additionally, age, level of education, and organizational position were correlated with distributive justice and procedural justice. Therefore, we decided to examine the effects of these demographic variables in the subsequent analyses.

**Table 4:** Correlation analysis (*n* = 747).

	**M ± SD**	**1**	**2**	**3**	**4**	**5**	**6**	**7**
1.Gender	1.44 ± 0.50	1						
2.Age	2.14 ± 1.00	−0.194^**^	1					
3.Level of Education	2.53 ± 1.06	−0.074^*^	0.207^**^	1				
4.Position	1.52 ± 0.79	−0.087^*^	0.246^**^	0.303^**^	1			
5.DJ	1.48 ± 0.50	0.001	0.081^*^	−0.126^**^	−0.089^*^	1		
6.PJ	1.50 ± 0.50	0.023	−0.029	0.099^**^	0.049	−0.015	1	
7.POB	6.16 ± 2.32	0.016	−0.018	−0.043	−0.002	0.079^*^	0.319^**^	1

* p < 0.05;

** *p < 0.01, two tailed test. Gender: 1 = male, 2 = female. Age: 1 = under 25 years old, 2 = 25–34 years old, 3 = 35–44 years old, 4 = 45–54 years old, 5 = over 55 years old. Level of education: 1 = under or junior high schools, 2 = high schools, 3 = junior college degree, 3 = bachelor degree, 4 = master and Ph.D. degree. Position: 1 = ordinary employees, 2 = first-line managers, 3 = middle managers, 4 = senior managers. DJ: 1 = distributive injustice; 2 = distributive justice. PJ: 1 = procedural injustice; 2 = procedural justice*.

#### Hierarchical regression analyses

We preformed a hierarchical regression analysis for the effect of each predictor on the outcome variable POB of employees. Our goal was to determine if the hypothesized variables added a unique contribution in the prediction of the criterion above and beyond the control variables. As such, we first entered the control variables. Second, we entered the distributive justice. Next, we entered the procedural justice. To control for potential demographic effects, we included age, gender, highest level of education and organizational position as control variables.

As shown in Table 5, the individual characteristics did not account for the variance in POB, and distributive and procedural justice predicted 10% of the variance in POB of employees. Excluding the effects of distributive justice, the strongest predictor of POB was procedural justice which means that the higher the perceptions of procedural justice, the more POB employees performed.

**Table 5:** Hierarchical regression analyses (*N* = 747).

**Predictors**	**Standard regression coefficient**		
	**Step 1**	**Step 2**	**Step 3**
Gender	0.006	0.008	0.020
Age	−0.015	−0.004	−0.022
Level of Education	−0.052	−0.064	−0.028
Position	0.016	0.008	0.024
Distributive justice		0.099^*^	0.098^**^
Procedural justice			0.317^***^
Adjusted *R*^2^	−0.003	0.005	0.103
Δ*R*^2^	0.003	0.009	0.100
*F*_(7, 747)_	0.521	1.75	14.47^***^

* p < 0.05;

** *p < 0.01*,

*** *p < 0.001. two tailed test. ΔR^2^ = Change in R^2^*.

#### Examining the effectiveness of experimental operation

**Figure 2:**
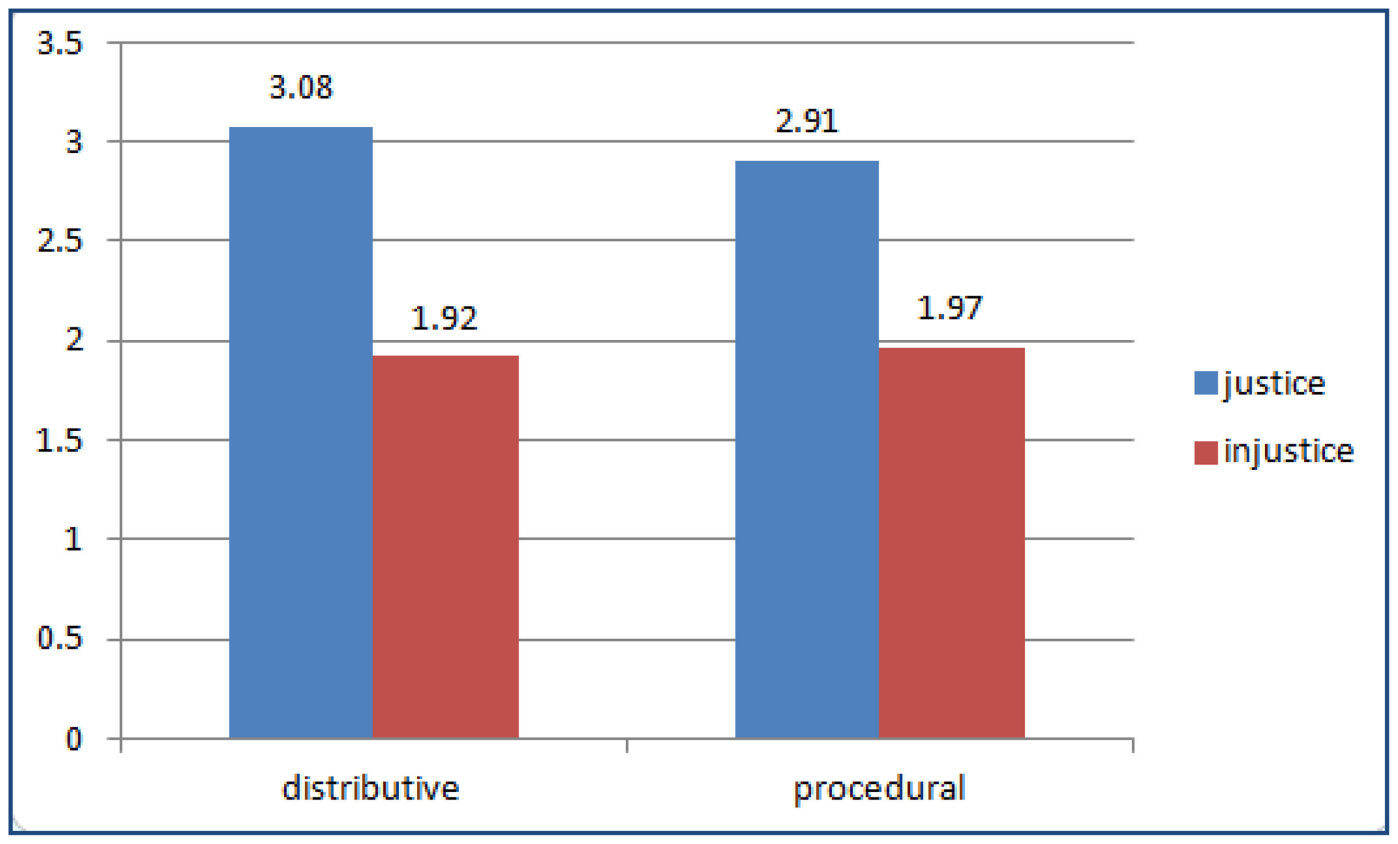
Experimental operational effectiveness of dependent variable.

Before verification of the research hypothesis, the discriminability of dependent variable was examined through the pretest. As shown in the Figure 2, the results of *t*-test indicated that the experience of distributive justice was more frequent than the experience of distributive injustice to the subject under the situation of distributive justice (M_distributive justice_ = 3.08, M_distributive injustice_ = 1.92, *t* = 97.10, *P* < 0.001). The experience of procedural justice was also more frequent than the experience of procedural injustice to the subject under the situation of procedural justice (M_procedural justice_ = 2.91, M_procedural injustice_ = 1.97, *t* = 32.87, *P* < 0.001). Hence, OJ experienced by the subjects was equal to the experimental orientation, and the experiment had obvious discriminability.

**Figure 3:**
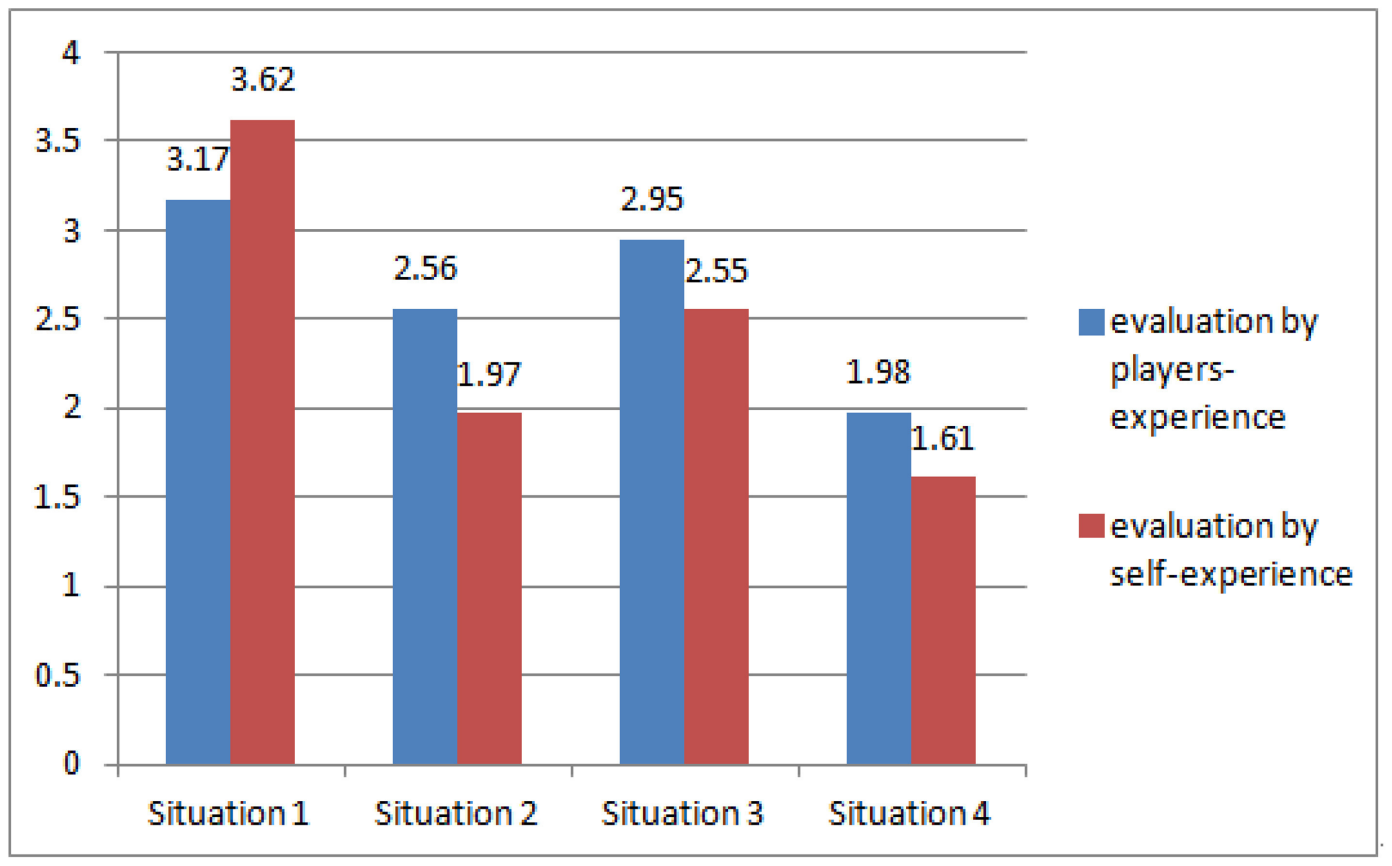
The effectiveness of evaluation by situation and evaluation by self-experience.

The effectiveness of evaluation by situation and evaluation by self-experience were also examined. As shown in the Figure 3, the results of the *t*-test indicated that the difference in OJ evaluation between others in the situation and themselves in the situation was obvious. Thus, (Situation 1: M_peer assessment_ = 3.17, M_self−evaluation_ = 3.62, *t* = 3.20, *P* < 0.01; Situation 2: M_peer assessment_ = 2.56, M_self−evaluation_ = 1.97, *t* = 2.58, *P* < 0.01; Situation 3: M_peer assessment_ = 2.95, M_self−evaluation_ = 2.55, *t* = 3.55, *P* < 0.001; Situation 4: M_peer assessment_ = 1.98, M_self−evaluation_ = 1.61, *t* = 3.12, *P* < 0.01). The results indicated a subject effect. Therefore, the effect of the independent variable on two response variables should be further examined.

#### Full model analysis

As presented in Table 6, we used multivariable variance analysis to examine the hypothesis proposed in this study. The results showed that distributive justice and procedural justice had a significant effect on employee POB, both in situational or self-experienced behavior. Furthermore, the interactions between distributive justice and procedural justice are significant (*p* < 0.001).

**Table 6:** Full model analysis.

	**Evaluated by situation**				**Evaluated by self-experience**			
	**Wilks'∧**	**F**	**Df**	**P**	**Wilks'∧**	**F**	**df**	**P**
Distributive justice (A)	0.98	5.19	4	0.003	0.98	5.25	4	0.002
Procedural justice (B)	0.88	36.15	4	0.000	0.88	36.21	4	0.000
A × B	0.98	6.97	4	0.000	0.98	6.73	4	0.000

#### Analysis of the effect of distributive justice and procedural justice on POB and negative organizational behavior

The full model test to experimental hypothesis was general, which made the analysis of the effects of independent variable on dependent variables difficult to conduct. Hence, a one-way ANOVA was adopted in this study to verify H4.

**Figure 4:**
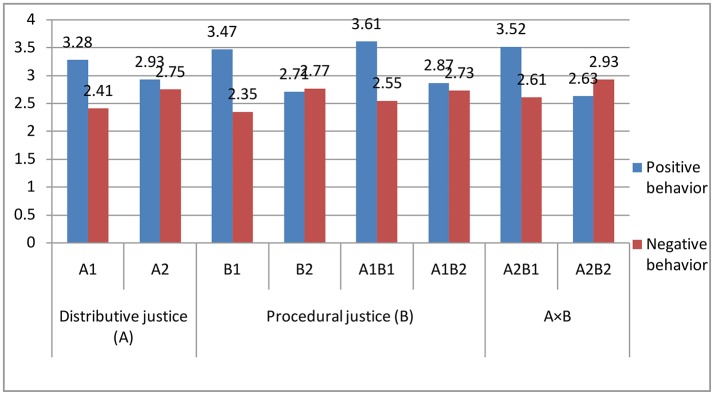
Effects of Distributive Justice and Procedural Justice to Positive/Negative Organizational Behavior.

As shown in Table 7 and Figure 4, employee positive behavior under the situation of distributive justice was more common than under the situation of distributive injustice (M_distributive justice_ = 3.28, M_distributive injustice_ = 2.93, *F* = 9.67, *P* < 0.001). Employee negative behavior under the situation of distributive justice was less than the behavior under the situation of distributive injustice (M_distributivejustice_ = 2.41, M_distributiveinjustice_ = 2.75, *F* = 7.87, *P* < 0.001). Employee positive behavior under the situation of procedural justice was more common than that under the situation of procedural injustice (M_proceduraljustice_ = 3.47, M_procedural injustice_ = 2.71, *F* = 127.35, *P* < 0.001). Employee negative behavior under the situation of procedural justice was also less than that in the situation of procedural injustice (M_procedural justice_ = 2.35, M_procedural injustice_ = 2.77, *F* = 19.17, *P* < 0.001). This outcome further confirmed that the distributive justice and procedural justice had significant effects on the pros and cons of employees' POB. The difference of interaction level between the distributive justice and procedural justice was significant (*P* < 0.05). In terms of the positive behavior, the diversity among A1B1, A2B1, and A1B2 showed that A2B2 was more obvious. In terms of the negative behavior, the diversity among A1B1, A2B1, and A1B2, A2B2 was less obvious. The difference between these situations was also obvious. Hence, procedural justice had a significant effect on POB, while distributive justice had a significant effect on NOB. In addition, from R^2^ in Table 7, the explanation of distributive justice and procedural justice effects on POB was 13.8% and for the NOB, the rate was only 3.6%. Therefore, OJ had greater effect on POB than that on the NOB. Therefore, H4 could be verified.

**Table 7:** Effects of distributive justice and procedural justice on positive/negative organizational behavior.

	**Positive behavior**					**Negative behavior**			
		**M ± SE**	**Df**	**F**	**P**	**M ± SE**	**df**	**F**	**P**
Distributive justice (A)	A1	3.28 ± 0.05				2.41 ± 0.05			
	A2	2.93 ± 0.04	1	9.67	0.003	2.75 ± 0.06	1	7.87	0.005
Procedural justice (B)	B1	3.47 ± 0.05				2.35 ± 0.04			
	B2	2.71 ± 0.04	1	127.35	0.000	2.77 ± 0.06	1	19.17	0.000
	A1B1	3.61 ± 0.06				2.55 ± 0.05			
A × B	A1B2	2.87 ± 0.05	1	2.37	0.041	2.73 ± 0.06	1	2.75	0.021
	A2B1	3.52 ± 0.06				2.61 ± 0.05			
	A2B2	2.63 ± 0.07				2.93 ± 0.09			
R^2^	0.138					0.036			

*A1, Distributive justice; A2, Distributive injustice; B1, Procedural justice; B2, Procedural injustice*.

In addition, the results also showed that distributive justice and procedural justice explained 13% variation of POB of employee, and explained only 3.6% variation of NOB. Therefore, OJ had greater effect on POB than NOB.

## General discussion

The correlation analysis showed that overall POB of employee has a significant correlation with organizational justice in manufacturing-type enterprises. The results also suggested that all dimensions of POB of employee were significantly related to distributive justice and procedural justice (*r* > 0.3, *P* < 0.001). Multiple regression analysis showed that OJ has a significant effect on POB of employees, and procedural justice and distributive fairness had significant positive effects on all dimensions of POB of employees. In other words, OJ is a positive predictor of POB of employee.

The situational experiment also confirmed the significant effects of procedural and distributive justice on positive and negative justice, as well as their interaction. The results confirmed that distributive and procedural justice had a significant effect on POB from the pros and cons of employee's POB. Many researches demonstrated that OJ had the remarkable two-way effect on employees' behavior. Specifically, organizational justice would boost employees' positive attitudes and behavior related to organizations such as improving employee job satisfaction (Tammy et al., 2010; Yijuan et al., 2011; Khan et al., 2015), organizational commitment (Ölçer, 2015; Sökmen and Ekmekçioglu, 2016), organizational productivity (Imran et al., 2015), organizational citizenship behavior (Karriker and Williams, 2009; Tziner and Sharoni, 2014; Gurbuz et al., 2016) and job performance (Walumbwa et al., 2009).

But if employees feel organizational injustice, they will display negative attitudes and behavior outcomes (Adams, 1965; Greenberg, 1990, 1993, 2001; Li and Shi, 2003; Pi, 2006; Ceylan and Sulu, 2011; Mingzheng et al., 2014; Chih et al., 2016). These results were in line with previous studies. Further, this research examined different directions of procedural and distributive justice on POB. Procedural justice is more likely to trigger POB of employees, whereas distributive injustice tended to result NOB. OJ also had greater effects on the POB of employees than NOB.

Further, this research examined different direction of procedural and distributive justice on POB. Procedural justice is more likely to trigger POB of employees, whereas distributive injustice tended to trigger NOB. OJ also had greater effects on the POB of employees than NOB. Therefore, future researchers should further analyze the relationship of these related factors with OJ or the POB of employees.

In addition, the findings revealed the remarkable result that the explanatory power of OJ theory to employees' POB was weak, which may have something to do with the effects of the Chinese traditional culture. In China, people pay attention to humanity, face, and euphemistical interpersonal association. In addition, people think highly of collectivity, collective honor, connotation, and great harmony. Under the cultural background of harmony (Yang, 1988; Wang and Zheng, 2005), the consciousness of dealing with affairs according to justice, procedure, social contract, and regulation is weaker than among Western people. In China, social relation network, implicit rules, and the way of saving the nation by curve may be more effective in social and enterprise management. Thereby, the consciousness of justice is weak in the mind of enterprise employees. The relationship comes down in one continuous line with the discovery that Chinese enterprise employees attach considerable attention to seeking harmonious interpersonal relations. Given the special cultural background in China, employee ownership of organizational achievement and reputation, organizational belongingness, sense of worth and pride based on the organization, sense of cohesiveness formed by organizational ideals and organizational support have far more significant effect on employees' POB than OJ. Hence, future replication studies in other contexts are needed to verify the findings of this study and consider the relationship between these factors described above and POB of employees.

## Strengths and limitations

A key strength of the present research is that we used a newly explicit definition of POB: an organizational behavior of employees would be beneficial to organization. The POB of employee is mainly composed of devoted, responsible, active, innovative, helping, and harmonious behavior. It is important to emphasize that this framework of POB can be measureable, assessable, controllable and changeable and it was conformed to the Chinese cultural background. Future research should investigate whether this definition of POB can conduct on other specific cultural, industrial and regional group.

The study has several limitations. First, all the measures in this study draw on self-reported data of individuals' attitudes and perceptions. Although we have used the pre-control method, such as ensuring the anonymity of respondents, designing the response questionnaire as a combination of self and peer evaluation and conducting the investigation in different periods, it may lead to common method variance that would has a negative impact on the reliability of the obtained results. We would encourage future researches to use longitudinal design to make causal statement address any concerns related to causal relationships, and also collect multiple data to measure the behavioral outcomes of POB. This would strengthen the research design and enhance the reliability of the results.

Second, the present study adopted two-factor models to analyze organizational justice because it was regarded as the most common model. However, it is suggested that for better understanding of OJ, interactional justice and informational justice can be included into the framework of OJ. Future researchers can analyze the effect of four dimensions of OJ on the POB of employees in detail.

Third, the experimental study used four different stimulus stories as materials to analyze the relationship between OJ and POB of employees, including devoted, responsible, active, innovative, helping and harmonious behavior. Although these stimulus stories were considered to be effective and reliable because of the connotation of these stories contains every facet of POB of employees (e.g., “striving for the best,” “working hard,” and “helping others”), we did not adequately measure other facets of POB of employees except for “active behavior”. Therefore, future research should address this issue by using diverse and complete materials and devising more elaborate procedures to examine the effect of OJ on POB of employees.

## Theoretical and practical implications

The findings of this study have many theoretical and practical implications for researchers and managers. From the theoretical perspective, firstly, the current research contributes to the existing literature by empirically investigating and validating relationships between organizational justice and POB. The obtained results of research demonstrate that the direct and positive relationships between organizational justice and POB are all statistically significant. And it reveals that procedural justice has a more powerful effect on POB of employees, whereas employees' NOB is more strongly influenced by distributive injustice. Moreover, the results of the path analysis show that organizational justice has stronger indirect impact on POB of employees than direct effects. Based on the findings of current research, researchers could extend the results of this study by considering other variables, in order to better comprehend and generalize the results of this study. Furthermore, researchers could consider the effect of economic situation on organizational outcomes. Specifically, whether people have stronger feelings of injustice during economic downturn and to what extent the economic crisis negatively affect employees' emotional and behavioral outcomes for organizations.

In addition, the current study distinguished that two dimensions (distributive and procedural justice) of OJ have distinct influence to employees' organizational behavior. It suggested that distributive justice and procedural justice would trigger different behavioral aspects of employees, which may make a contribution to the previous knowledge about the theory of organizational justice.

From the practical perspective, employees of an organization will reflect positive behavior and productivity if they perceive their organization as fair and just in its procedures and distribution systems. Enhancing organizational justice results in improved outcomes from employees. Therefore, managers should make efforts to enhance the perceived organizational justice of employees to improve their POB. Furthermore, the findings suggest that procedural justice differ from distributive justice in effectiveness and direction of effect. It is important for organizational managers to consider when they formulate and implement justice strategies to influence employees' related attitudes and behaviors. They should ensure both processes are fair, transparent and just and distributions are equitable and reasonable. Therefore, managers are encouraged to have a comprehensive consideration to increase the POB of employees and to decrease the NOB of employees, due to the employees' negative perception about distributive injustice.

To conclude, this current study contributes to the literature in the following ways. First, the current study offers a new perspective about POB of employees, including devoted, responsible, active, innovative, helping, and harmonious behavior. Second, this study confirms past findings by showing organizational justice has a significant impact on POB of employees. Finally, the study contributes to our understanding that two forms of organizational justice have different influences on employees' organizational behavior. In other words, procedural justice significantly influenced POB of employees, and distribution injustice significantly influenced NOB.

## Conclusion

Employees' POB has an obviously positive relation with OJ in the manufacturing-type enterprises. OJ clearly indicates the positive prediction on POB. Situational experiments have further confirmed that the main effect of procedural and distributive justice on POB and NOB is obvious, and there is frequent interaction between them. In addition, the influence orientation and the effectiveness between procedural and distributive justice also differ. In other words, procedural justice is prone to result in POB and distributive justice has a significant effect on negative organizational behavior. Furthermore, path analysis suggested that OJ has more indirect effects on POB than direct effects, which could probably because the effects of other mediating variables.

## Ethics statement

The study was carried out in accordance with the recommendations of “the University of Southwest's Human Research Ethics Committee” with written informed consent from all subjects. All subjects gave written informed consent in accordance with the Declaration of Helsinki. The protocol was approved by the “the University of Southwest's Human Research Ethics Committee.”

## Author contributions

The current research was carried out in collaboration between all authors. XP designed the study, analyzed the data and wrote the framework of the manuscript. MC analyzed the literature and wrote the first draft of the manuscript. ZH and WB conducted the literature research and carried out experimental process. Finally, XP and MC revised and perfected the manuscript. All authors read and approved the final manuscript.

### Conflict of interest statement

The authors declare that the research was conducted in the absence of any commercial or financial relationships that could be construed as a potential conflict of interest.

## Appendix

**Scene 2:** A1B2 (distributive justice × procedural injustice)

**(Story)**

Senior managers of a company intend to give a large amount of bonus for employees. *Not only the distributive standard is not known by* employees, *but also the evaluation process is not transparent. Even they don't know how their evaluation score is calculated by* company. *The result is not shown publicly. No reason for complaint if mistakes exist. Finally, zhangsan obtained a bonus. Later he learned that his bonus roughly consistent with his income*.

(Instructions) Please answer the following questions based on your real thoughts and physical truth according to the situational story. When answering the questions, mark directly on the selected option (5 = Absolutely agree, 4 = Partly agree, 3 = A little bit agree, 2 = Partly disagree, and 1 = Absolutely disagree).

Absolutely disagree—Absolutely agree

(1) Based on the bonus distributive procedure mentioned above, Zhangsan will think it is fair and he will do his work actively.1 2 3 4 5

(2) Based on the bonus distributive procedure mentioned above, if we were employees in the company, we would also feel it is fair and do our work actively.1 2 3 4 5

(3) Based on the bonus distributive procedure mentioned above, Zhangsan will feel it is unfair. He will slow down, be absent, not obey the arrangement in his work, or even resign. 1 2 3 4 5

(4) Based on the bonus distributive procedure mentioned above, if we were employees in the company, we would also feel it is unfair. We would slow down, be absent, not obey the arrangement in our work, or even resign. 1 2 3 4 5

**Scene 3:** A2B1 (distributive injustice × procedural justice) **(Story)**

Senior managers of a company intend to give a large amount of bonus for employees. They formulate the standards and organize multi-level managers and representatives of employees to have a discussion. After discussion, the distributive standard is determined preliminarily. Then, the standard is shown publicly to collect opinions from ordinary employees until all staves approve the standard. Based on the decision without objection, the Human Resources Department evaluates every employee according to the distributive standard and personal job performance. The result is shown publicly for correction if mistakes exist. According to the distributive arrangement and personal job performance, ZhangSan got the lowest score, while did not obtain the minimum bonus.

(Instructions)Please answer the following questions based on your real thoughts and physical truth according to the situational story. When answering the questions, mark directly on the selected option (5 = Absolutely agree, 4 = Partly agree, 3 = A little bit agree, 2 = Partly disagree, and 1 = Absolutely disagree).

Absolutely disagree—Absolutely agree

(1) Based on the bonus distributive procedure mentioned above, Zhangsan will think it is fair and he will do his work actively.1 2 3 4 5

(2) Based on the bonus distributive procedure mentioned above, if we were employees in the company, we would also feel it is fair and do our work actively. 1 2 3 4 5

(3) Based on the bonus distributive procedure mentioned above, Zhangsan will feel it is unfair. He will slow down, be absent, not obey the arrangement in his work, or even resign. 1 2 3 4 5

(4) Based on the bonus distributive procedure mentioned above, if we were employees in the company, we would also feel it is unfair. We would slow down, be absent, not obey the arrangement in our work, or even resign. 1 2 3 4 5

**Scene 4:** A2B2 (distributive injustice × procedural injustice)

**(Story)**

Senior managers of a company intend to give a large amount of bonus for employees. Not only the distributive standard is not known by employees, but also the evaluation process is not transparent. Even they don't know how their evaluation score is calculated by company. The results of bonus distribution are not shown publicly. Even if the results are wrong, the senior managers don't allow you to appeal freely. Finally, zhangsan obtained a bonus. Later he learned that his bonus did not consistent with his income. Compared with him, those who did less job, created lower performance, and contributed less, eventually obtained more bonuses.

(Instructions)Please answer the following questions based on your real thoughts and physical truth according to the situational story. When answering the questions, mark directly on the selected option (5 = Absolutely agree, 4 = Partly agree, 3 = A little bit agree, 2 = Partly disagree, and 1 = Absolutely disagree).

Absolutely disagree—Absolutely agree
(1) Based on the bonus distributive procedure mentioned above, Zhangsan will think it is fair and he will do his work actively.1 2 3 4 5
(2) Based on the bonus distributive procedure mentioned above, if we were employees in the company, we would also feel it is fair and do our work actively. 1 2 3 4 5
(3) Based on the bonus distributive procedure mentioned above, Zhangsan will feel it is unfair. He will slow down, be absent, not obey the arrangement in his work, or even resign. 1 2 3 4 5
(4) Based on the bonus distributive procedure mentioned above, if we were employees in the company, we would also feel it is unfair. We would slow down, be absent, not obey the arrangement in our work, or even resign. 1 2 3 4 5
